# 

**Published:** 1898-02

**Authors:** 


### PERSONALS.

Dr. N. Rectenwald, of Pittsburg, has just added another beacon of joy in his family, the birth of a daughter, the fourteenth of his little ones, twelve of whom are living.

Dr. Leonard Pearson spent several days in January among the Farmers' Institutes.

Dr. A. J. Sheldon enters practice with Messrs. Lyman and Osgood, under the firm name of Lyman, Osgood & Sheldon, at 50 Village Street, Boston, Mass.

Drs. James A. and David Waugh, of Pittsburg, will give instructions to a number of horseshoers in that city this winter. The course will consist of a series of lectures and anatomical dissections and demonstrations.

Dr. Wm. H. Nice, of Germantown, Philadelphia, fills an appointed office in Union Lodge, F. and A. M., one of the oldest and richest lodges in the jurisdiction of Pennsylvania.

Dr. James A. Marshall has been elected Vice-President of the Philadelphia Turf Club.

Dr. George W. DeSwan, of Roxborough, holds the position of veterinarian to the Philadelphia Retail Grocers' Association.

Veterinarian D. Cumming, Inspector of the Bureau of Animal Industry at Port Huron, Michigan, was on the sick-list in January.

Dr. J. S. Alexander, of Evanston, Illinois, was a contributor to the Christmas number of the *Breeders' Gazette.*

Drs. Adams and Harger will again lecture to the horseshoers of Philadelphia during the coming months.

Veterinarian J. Aschmidt was appointed one of the Board of Examiners of horseshoers in Minnesota by the Governor.

Dr. W. L. Rhoads, of Lansdowne, Pa., met with an extremely narrow escape from injuries by a trolley-car. A thorough shaking up, some bruises, the destruction of his carriage, and injuries of a severe character to his horse completed the wreckage.

Dr. Robert Ward, of Baltimore, was a contributor to the holiday number of the *Horseshoers' Journal.*

Dr. Charles A. Gleason, of Rye, N. Y., has been elected President of No. 94 of the Master Horseshoers' Union.

Veterinary Surgeon John J. Fox, of New York City, has retired from the veterinary profession and adopted the vocation of undertaker and embalmer. Dr. John Dodd succeeds to his practice.

Dr. Emil Knight, of Rochester, N. Y., met with the sudden and sad loss of his mother, in January, from cerebral hemorrhage.

Dr. Patrick H. Mullowney, of Boston, has accepted an appointment in the Bureau of Animal Industry and has been assigned to duty in the quarantine service west of the Mississippi River.

Dr. Leslie J. Allen has been transferred from the meat-inspection service at Kansas City to the western quarantine division, and will be stationed at El Paso, Texas, from which post Dr. A. B. Morse was recently transferred to Clinton, Iowa, to take charge of the abattoir inspection at that point.

Dr. J. I. Gibson, of Dennison, has been reappointed State Veterinarian of Iowa by Governor Shaw.

Dr. C. F. Bell, of Kokomo, is a breeder of bloodhounds, and has recently been elected President of the American Bloodhound Association.

Dr. U. G. Houck, of the Bureau of Animal Industry, has been transferred at Chicago from the inspection service to microscopical work in the laboratory at that point.

Dr. N. A. Cohen has been elected Secretary-Treasurer of the Wildwood, N. J., fire company.

Dr. H. D. Fenimore, of Knoxville, Tenn., was a visitor during the holidays to the "City of Brotherly Love," including the JOURNAL office among his calls.

Dr. J. W. Dunham, formerly of Faribault, Minn., is now located at Lakota, North Dakota.

Veterinarian Wm. J. Waugh is now located with his company at Fort Ethan Allen, Essex Junction, Vt.

Dr. W. L. Labaw, formerly assistant of Lyman & Osgood, of Boston, has entered upon private practice in the city of Boston.

Dr. Leonard Pearson was a visitor to Boston on New Year's Day, spending some hours in clinical instruction at the Harvard Veterinary Department.

Dr. E. C. Muir, of the Veterinary Department of the University of Pennsylvania, is conducting a series of experiments in equine cathartics.

State Veterinarian Leonard Pearson was a visitor to Washington, D. C., in January, in the interests of the regulations of the Pennsylvania State Live-stock Sanitary Board, regarding cattle-traffic through the Keystone State.

Dr. A. J. Sheldon is now connected with the clincal staff of instructors of the Veterinary Department of Harvard University.

Prof. S. J. J. Harger, addressed the Students' Society in January at the Veterinary Department of the University of Pennsylvania, on " Infectious Abortion of Cattle."

Dr. E. G. Gilbert, of Pottstown, was a visitor to Philadelphia in January, on a mission relative to the regulations of the Pennsylvania State Live-stock Sanitary Board.

Dr. Leonard Pearson was in attendance upon the annual meeting of the State Board of Agriculture of Pennsylvania, at Harrisburg, in January, and also at the subsequent reception given this body by Governor Hastings.

Dr. L. A. Merillat, of the McKillip Veterinary College, was called as an expert in the second trial of A. L. Luetgert for murder, testifying that the disputed fragment of bone on which so much rests was a portion of the os navicula of the ox. This evidence was in conflict with the State's witnesses, who claimed it as a part of the head of a human rib.

Dr. H. A. Christmann, of Philadelphia, has received an appointment under the Bureau of Animal Industry, and has been assigned to duty at Milwaukee, Wis.

Dr. J. C. McNeil, of Pitsburg, is giving much attention to the examination of live-stock entering Pennsylvania to replenish our dairies under the regulations regarding tuberculosis.

Dr. Alex. Glass, of Philadelphia, is forming a reading-class in French.

Dr. Herbert Neher, of New York City, was on the sick-list and absent from duty the latter part of the old year.

Dr. Wm. Herbert Lowe, of Paterson, N. J., has placed upon the streets of that city a commodious ambulance, and in his noted generosity wants it to be known that it will be at the command of all his colleagues at any hour for the prompt conveyance of sick or disabled animals to any point.

### INTERESTING CORRESPONDENCE.

"Where ignorance is bliss, it is folly to be wise."

R———, Pa., Dec. 7th, 1897.

Dr. V——, V.S.:

Dear Sir Will you Please Give Me your Remedy For Lung Feaver as I Have Bin told you are verry Successful In The Treatment of Lung Feaver In Horses I would Be verry Thankfull If you Would Send Me your Remedy as I Have Some Cases That Die For Me & Especilly Where Their is a Tendency To Dropsey I Will Give you My Remedy For (Partuerenty Appolexia or Milk Feaver) as Some Call it) as I Loose Verry Few Cases.

R.—Tr. acconite

" Belladonna | . alternally Evry 2 Hours 20 Drops.

& when The The Feaver Subsides Give The usual Dose of (ammonia balerian N.

Yours in
.F. C. B.
Dr. G. C. B——, D.V.s.

Please Send as I am Many Miles From you & Cannot Do you Eny Hurt Mr. G.—— H—— told Me of your Successfull Treatment of Some western Horses of abraham C—— at Three Bridges N Y

G. C. B.

[Editor.—The above is an exact reproduction of a letter sent by one veterinarian to another, and should be a sufficient reply to those who still feel that there is no need of Boards of Veterinary Medical Examiners.]

